# A Miraculous Recovery

**Published:** 1885-01

**Authors:** 


### A MIRACULOUS RECOVERY.

In his interesting novel, "Martin the Foundling," Eugene Sue, the eminent French writer, gives the following account of a wonderful cure by natural agencies, and adds that he can vouch for it, as it was performed by his father, the late Dr. Sue. The patient, a wealthy Count, was so gratified at what he termed his "resurrection," that he designed a monument to the doctor, a reduced copy of which may be seen in the Royal Art School of Paris, to which it was bequeathed by Dr. Sue at his death.

"Let me tell you of a strange and almost miraculous fact, of which I was witness, and which will fully possess you with my thought. I was at the time in the service of an eminent physician, celebrated alike as a philosopher and a profound thinker. He was one day called upon to attend a wealthy patient, whom he found apparently expiring, exhausted by excessive pleasures of every description. His blood, vitiated and impoverished, circulated languidly in his almost dried-up veins, no longer as the fluid of life but as a fluid of death. The most eminent and skillful physicians had abandoned all hope of curing the unfortunate creature. The philosopher and profound thinker then called to mind those frightful and mysterious stories which tell of young and generous blood being transfused into the veins of some old men worn down by debauchery. Blood was not necessary, but that sanguinary fable put the philosopher upon the track of an admirable idea. Hangings of silk and gold, impregnated with unwholesome perfumes, covered the walls of the rich man's splendid room, and half darkened it. These hangings the skillful physician ordered torn down, and the healthy air and sun penetrated in every direction. The walls were then covered with green branches freshly torn from resinous and balsamic trees, abundantly exhaling those gases which render the air pure and life-sustaining. Then young, strong and healthy matrons gave their full bosoms to the expiring mouth of the dying man. Oh! miracle. Scarcely were the parched lips moistened with that regenerating beverage ; scarcely had the patient inhaled the salubrious and reviving air exhaled by the green branches which alone now surrounded his bed, before he seemed to revive and did revive. His impoverished and corrupted blood became renewed and invigorated. He lived ; and his safety cost neither blood nor tears. Pure and nourishing milk, some fresh green boughs, and the beneficent rays of the sun were the sole means of that wonderful cure."

